# Who’s afraid of the big bad wolf? Variation in the stress response among personalities and populations in a large wild herbivore

**DOI:** 10.1007/s00442-018-4174-7

**Published:** 2018-05-26

**Authors:** Nadège C. Bonnot, Ulrika A. Bergvall, Anders Jarnemo, Petter Kjellander

**Affiliations:** 1 Grimsö Wildlife Research Station, Department of Ecology, Swedish University of Agricultural Sciences, 730 91 Riddarhyttan, Sweden; 2 Department of Zoology, Stockholm University, 106 91 Stockholm, Sweden; 3 School of Business and Engineering, Halmstad University, P. O. Box 823, 301 18 Halmstad, Sweden

**Keywords:** Coping style, Docility, Predation risk, Glucocorticoid, Neonatal period

## Abstract

Faced with rapid environmental changes, individuals may express different magnitude and plasticity in their response to a given stressor. However, little is known about the causes of variation in phenotypic plasticity of the stress response in wild populations. In the present study, we repeatedly captured individual roe deer (*Capreolus capreolus*) from two wild populations in Sweden exposed to differing levels of predation pressure and measured plasma concentrations of stress-induced cortisol and behavioral docility. While controlling for the marked effects of habituation, we found clear between-population differences in the stress-induced cortisol response. Roe deer living in the area that was recently recolonized by lynx (*Lynx lynx*) and wolves (*Canis lupus*) expressed cortisol levels that were around 30% higher than roe deer in the human-dominated landscape free of large carnivores. In addition, for the first time to our knowledge, we investigated the stress-induced cortisol response in free-ranging newborn fawns and found no evidence for hypo-responsiveness during early life in this species. Indeed, stress-induced cortisol levels were of similar magnitude and differed between populations to a similar extent in both neonates and adults. Finally, at an individual level, we found that both cortisol and docility levels were strongly repeatable, and weakly negatively inter-correlated, suggesting that individuals differed consistently in how they respond to a stressor, and supporting the existence of a stress-management syndrome in roe deer.

**Electronic supplementary material:**

The online version of this article (10.1007/s00442-018-4174-7) contains supplementary material, which is available to authorized users.

## Introduction

Predation pressure is a major selective force shaping prey phenotypes, both directly (lethal effects) and indirectly (non-lethal effects), through the costs of anti-predator responses (Lima 1998; Preisser et al. 2005; Creel and Christianson 2008). As a consequence, to maximize individual fitness, prey have evolved physiological, behavioral and morphological responses to cope with these unpredictable and potentially deleterious predation-induced stressors (Lima 1998; Wingfield and Romero 2001). However, anti-predator responses generally incur costs in terms of somatic growth, body condition, reproduction or survival, so that the indirect effects of predation risk can be as great as, or even greater than, the direct effects (Boonstra et al. 1998; Preisser et al. 2005). For example, in the Yellowstone National Park, recent studies suggested that the reintroduction of wolves (*Canis lupus*) may have led to an increase in vigilance levels of elk (*Cervus elaphus*), a modification of their feeding behavior and nutritional condition, and an increase in mean progesterone concentrations in females, leading to a decline in calf production (Creel et al. 2007; Christianson and Creel 2010).

Although anti-predator responses may evolve over evolutionary time through natural selection, animals can also plastically adjust their responses to environmental changes experienced within their lifetime. For example, predator cues may drive ontogenetic development of inducible defenses (sensu Tollrian and Harvell 1999) in morphological structures and behaviors. The ability to produce alternative phenotypes in response to environmental change may allow organisms to maximize fitness by matching their phenotype to the prevailing environmental conditions (Scheiner 1993). The magnitude and the plasticity of the stress response may vary depending on the individual’s state and/or phenotype (e.g. sex, age or body condition), or in relation to the individual’s sensitivity to stress or personality (Koolhaas et al. 1999; Novais et al. 2017). These among-individual differences are generally considered within the framework of coping styles, reflecting how an individual deals with a stressful or challenging situation along a reactive-proactive gradient (Koolhaas et al. 1999). Because behavioral variations in the stress response are often mediated by neuroendocrine pathways, both physiological and behavioral responses are important components for defining an individual’s coping style (Koolhaas et al. 2007; Réale et al. 2010). For example, strains of zebra finches that were artificially selected for their endocrine hormonal responsiveness differed in their willingness to take risk and their exploratory behavior (Martins et al. 2007).

Habituation is a particular case of plasticity, resulting in dampening of the stress response following repeated exposure to a mild stressor (Rankin et al. 2009). Because stress responses to both direct reactive and indirect anticipatory stressors (see more details in Boonstra 2013) are costly in terms of energy, potentially inducing long-term deleterious effects on individual fitness (Bonier et al. 2009; Clinchy et al. 2013), habituation prevents unnecessary overreaction to non-threatening stimuli. In risky environments, where predators are relatively abundant and prey are regularly exposed to predators or cues of their presence (e.g. odour), the predator-induced stress response may become attenuated through habituation. However, when submitted to novel stressors, the response of habituated animals is generally more acute compared to non-habituated animals (i.e. sensitization, Romero 2004). Therefore, the level of the stress-induced response expressed by an individual depends on its previous experience in terms of exposure to stressors.

Sensitivity to stress and the adjustment of the stress response (e.g. through habituation) have both been related to the hypothalamic–pituitary–adrenal (HPA) axis (for more details, see for example Reeder and Kramer 2005). Indeed, when faced with a stressor, animals instantaneously respond physiologically through the release of glucocorticoids (i.e. steroid hormones) by the HPA axis. In the context of predation, glucocorticoids, such as cortisol, allow animals to react efficiently and express an appropriate anti-predator behavior to maximize survival (Wingfield and Romero 2001). Although cortisol is involved in numerous metabolic functions in both basal and stress-induced contexts, prolonged exposure to high levels of cortisol due to continued or repeated exposure to stressors may be deleterious (Bonier et al. 2009; Clinchy et al. 2011; Boonstra 2013). For example, high predator-induced cortisol concentrations in snowshoe hares (*Lepus americanus*) resulted in a decrease in their reproductive performance (Sheriff et al. 2009). However, such examples of a long-term effect of predation risk on fitness components mediated by chronic stress are rare (see also Clinchy et al. 2004; McCormick 2009), and not always supported (e.g. Creel et al. 2009). For example, in Yellowstone National Park, the wolf-induced risk effects on elk reproduction appeared to be mediated more by nutritional costs associated with the behavioural modification of elk (predator-sensitive food hypothesis) than by chronic activation of the cortisol stress response (predation stress hypothesis) (Creel et al. 2009).

Exposure to stress early in life, including prior to birth through maternal transmission, can also induce permanent changes in offspring physiology, behavior and/or morphology (Love et al. 2013). Indeed, the stress level of gestating or lactating mothers has been shown to influence the behavior and stress response of their offspring (Hayward and Wingfield 2004; Petelle et al. 2017). For neonates, stress during early life development can have negative effects, notably on neuronal growth (Lupien et al. 2009). In many mammalian species, neonates have a rather poor ability to respond to stressors (i.e. hypo-responsiveness), which is thought to be an adaptation to protect them from the deleterious effects of stress during the initial rapid growth phase (De Kloet et al. 1988; Romero 2004).

While the behavioral and physiological components of stress have been well studied under controlled laboratory conditions, much less is known about free-ranging animals in their natural environment (Reeder and Kramer 2005; Newman et al. 2013). In this study, we measured the stress-induced plasma cortisol and behavioral responses of roe deer (*Capreolus capreolus*) in two populations exposed to contrasted environments, notably in terms of predation risk. Both populations were free of large natural predators after their eradication in the beginning of the 19th century (Ripple et al. 2014). However, whereas this is still the case in the first population, located in a human-dominated environment in south-eastern Sweden, the second population in south-central Sweden now coexists with both lynx (*Lynx lynx*) and wolf (*C. lupus*), provoking a considerable decline in roe deer density (Andrén and Liberg 2015). As a consequence, we expected those roe deer facing the return of large natural predators to respond more strongly to a stressful event than roe deer living in the human-dominated area (**H1**). Because we captured individuals multiple times within their lifetime, we were able to investigate how the deer habituated to the stress of capture over both the short-term (within year) and long-term (among-year) by studying attenuation in stress responsiveness. In addition, for the first time to our knowledge in a free-ranging mammal, we investigated variation in the physiological stress response at capture in neonates. Because of the expected hypo-responsive period, we predicted that neonates should exhibit a lower initial stress-induced cortisol response compared to adults (**H2**). Finally, as previous studies have suggested that alternative coping styles exist in roe deer (Debeffe et al. 2014; Bonnot et al. 2015; Monestier et al. 2015), we expected to observe consistent among-individual variation in how deer responded to capture, describing a proactive–reactive gradient (**H3**). To test this hypothesis, we investigated individual repeatability in the level of cortisol and docility during handling, as well as the relationship between these metrics that index the physiological and behavioral components of the stress response. We assumed that proactive individuals would exhibit lower levels of cortisol and behavioral responsiveness (i.e. be more docile), whereas reactive individuals would have higher cortisol levels and demonstrate stronger behavioral responsiveness (i.e. be less docile).

## Methods

### Study areas

We used long-term data collected from two populations of European roe deer which have been intensively monitored since 1973 and 1988, respectively: Grimsö Wildlife Research Area (GWRA) located in south-central Sweden (59°40′N, 15°25′E) and Bogesund located in south-eastern Sweden (59°23′N, 18°15′E). GWRA and Bogesund have contrasted landscapes differing in the levels of human disturbance and predation pressure, resulting in different mortality patterns for roe deer (see Online Resource 1). At GWRA, the landscape is covered by 73% intensively managed mixed conifer forest, 19% bogs and 2% meadows and farmlands (Rönnegård et al. 2008). Human pressure is low, with a mean human density of around 17 individuals/km^2^. Hunting occurs from September to February. In recent decades, roe deer density has decreased considerably (from 106 to 8 deer/1000 ha between 1984 and 2016; SITES unpublished data) following the return of lynx in 1996 and wolf in 2003 (Wabakken et al. 2004; Liberg and Andrén 2006). Predation is the main cause of mortality for adult roe deer in GWRA (i.e. 59% of all mortality events recorded over a 5 years period, see Online Resource 1 for more details). The lynx is the main predator of roe deer in GWRA (Andrén and Liberg 2015), accounting for 37% of mortality events in adults and 22% in neonates. However, red fox (*Vulpes vulpes*) is the major predator of newborn fawns (Jarnemo and Liberg 2005), accounting for 29% of mortality during summer (Online Resource 1).

Bogesund is a fragmented landscape covered by 65% forest, 10% bogs and 25% farmlands (Kjellander et al. 2006). Human pressure is relatively high, with a mean human density of around 200 individuals/km^2^ and recreational activities throughout the year. Hunting occurs from September to January and represents the main mortality cause for adult roe deer (see Online Resource 1). Since 2008, roe deer density has been relatively stable at around 100 deer/1000 ha. No large predators are permanently resident in Bogesund. In contrast, red fox density, although largely variable among years, is generally higher than in GWRA (0.19 ± 0.11 in Bogesund vs 0.08 ± 0.03 in GWRA) and can markedly affect survival of neonates during their first weeks of life (Online Resource 1; Jarnemo and Liberg 2005).

### Roe deer capture and data collection

#### Winter capture of roe deer of at least 5-months old

Each winter, between November and March, roe deer of at least 5-months, and up to 12-years old, were caught in wooden box traps at both study sites. Since 2009, during winter, we captured 301 individuals at GWRA (53% females) and 243 at Bogesund (59% females). The traps are generally activated at sunset and checked the next morning (between 7:00 and 10:00), facilitating capture during crepuscular hours and nighttime when roe deer are the most active (Pagon et al. 2013). Each animal was individually marked, sexed, weighed and aged. Since 2011, blood samples were collected from the jugular vein (9–40 ml) using 0.8 × 40 mm needles (Vacuette, Greiner Bio-One GmbH, Austria) at each first annual capture of an individual, when possible, and up to 5 times more during the same winter (see Table 1). As the marking procedure usually lasted 10–15 min, the blood cortisol concentration indexed the stress-induced hormonal response to capture (Reeder and Kramer 2005). In total, we assayed blood cortisol levels for 131 roe deer (Table 1).

**Table 1:** Summary of data collected in the two study areas (GWRA and Bogesund) to assess cortisol and docility responses in roe deer of more than 5-months of age (winter) and neonates (summer) to repeated capture

	Study area	Number of observations	Number of individuals	Number of observations per individual per year (mean and range)	Number of recaptures per individual per year (mean and range)
Cortisol sample					
Winter captures	GWRA	236	131	1.3 [1–6]	2.2 [1–16]
	Bogesund	63	49	1.0 [1–1]	2.1 [1–10]
Summer captures	GWRA	113	76	1.5 [1–3]	2.2 [1–4]
	Bogesund	63	41	1.5 [1–3]	2.1 [1–3]
Docility score					
Winter captures	GWRA	924	301	1.8 [1–9]	1.9 [1–16]

Since 2009, in GWRA only, the behavioral responsiveness to handling, i.e. docility, was also assessed for each individual by a single experienced handler. The occurrence and intensity of several behavioral components (notably, vocalization, struggling and kicking) were used to index docility during manipulation (see more details in Debeffe et al. 2015). The resulting handling score ranged from 0 to 4, with 0 indicating docile individuals, while 4 indicates non-docile, highly stressed individuals. Although the absolute value of the score represents a relative judgement, such behavioral ranking methods are commonly employed to quantify docility in mammals (e.g. Réale et al. 2000; Petelle et al. 2017). We measured the docility score for 301 individuals (GWRA only, Table 1) at each first annual capture of an individual, when possible, and up to 8 times more during the same winter. Individuals recaptured at short intervals (e.g. within a week) were generally released directly without handling so that the number of recaptures per individual per year may differ from the number of samples per individual per year (Table 1). A given individual may also be captured several times over different winters (between 1 and 9 different winters of capture). We assume that there was no consistent bias in the time a given individual spent in the trap over multiple capture events which could have influenced its stress response.

#### Summer capture of neonates

Since 2013, we collected blood samples from newborn fawns, aged between 0 and 22 days old, during the summer fawning season (from May to July; 48% females). Each neonate was individually marked, sexed, weighed, aged (using the state of the umbilical cord and behavior at marking, following Jullien et al. 1992) and equipped with an expandable VHF-collar (Followit, Sweden; collar weight 65–70 g, < 5% body mass). Blood was collected from the brachial vein in 4 ml tubes using 21-gauge 0.7 × 40 mm needles (Vacuette, Greiner Bio-One GmbH, Austria). The marking procedure was carried out in proximity to the bed site and usually lasted 10–15 min. After marking, the neonate was replaced in its bed site. All procedures were approved by the Ethical Committee of Animal Experiments in Uppsala, Sweden (permits C302/12 and C289/09).

### Enzyme immunoassay

The blood samples were centrifuged (822×*g*, 10 min) within 3 h of collection to separate plasma which was first stored at − 20 °C and then at − 80 °C until analysis. Plasma cortisol concentrations were determined using an automated solid-phase enzyme-labeled chemiluminescent competitive immunoassay on Immulite 2000 (Siemens Healthcare Diagnostics, Erlangen, Germany) using reagents from Siemens Healthcare Diagnostics. The detection limit of plasma cortisol concentration was 10 nmol/l. Intra- and inter-assay coefficients of variation were 7.3 and 8.6%, respectively. We measured the cortisol response for 180 individuals older than 5 months of age (131 in GWRA and 49 in Bogesund) and 117 neonates (76 in GWRA and 41 in Bogesund; see Table 1).

### Statistical analysis

In this study, we investigated the stress responses of roe deer to capture in both neonates and deer of more than 5-months old independently. First, we investigated the physiological (cortisol) and behavioral (docility) responses in deer of more than 5-months old roe to handling stress. Because intrinsic factors may modulate the response of an individual to a given stressor, we controlled for the effects of sex and age (4 classes: juvenile of 5–10 months old, yearling of 1.5 years old, adult of 2.5–6.5 years old and old animals of 7.5–12.5 years old) in the analyses. To investigate habituation to repeated capture events over both the short and long-term, we determined the number of captures experienced by each individual within a year (Ncaptures, short-term habituation) and the number of different winters during which each individual was captured (Nwinters, long-term habituation). That is, we assumed that an individual will react differently when captured for the very first time within its lifetime (Ncaptures = 1, Nwinters = 1), compared to the first capture event of the year during a subsequent winter (Ncaptures = 1, Nwinters > 1). Because we did not expect long-term habituation to be governed by a linear effect, we included Nwinters as a categorical variable with three modalities: first winter of capture (1st winter), second and third winters of capture (2nd–3rd winters) and more than three winters of capture (> 3 winters). We chose these cut-off points to obtain comparable sample sizes among categories (that is, 457, 295 and 172 observations for the 1st, 2nd–3rd and > 3 winters, respectively). We analysed individual variation in the physiological and behavioral responses to stress by fitting two sets of linear mixed models, one to explain variation in cortisol levels and a second to explain variation in handling scores (docility). Because animals can habituate to repeated stressors over both the short- and long-term, we included the two-way interaction between the log-transformed number of captures experienced by an individual within a year (log(Ncaptures)) and the number of different winters of capture (Nwinters). To test for a difference in habituation between populations, we included the two-way interactions between log(Ncaptures) and study area and between Nwinters and study area. To account for intrinsic characteristics of individuals, we also included sex and age, as well as their two-way interactions with log(Ncaptures). Therefore, the most complex model explaining variation in cortisol levels included the four two-way interactions between log(Ncaptures) and, respectively, Nwinters, area, age and sex. The most complex model explaining variation in handling score was similar, except that we removed the effect of area, as docility was not assessed in Bogesund. Finally, we also included individual identity as a random factor on the intercept in all models to control for repeated observations of individuals. To control that the stress responses of deer did not vary in relation to time during the winter season, we performed a preliminary analysis using generalized additive models with the Julian date as a smoothing term. This analysis indicated no temporal variation in stress responses within the winter capture season (November–March), hence, we did not include time as an explanatory variable in our previous models. Finally, although we believe that the handling score describes a continuum in the behavioral response of roe deer to capture stress, for comparison, we also used ordinal mixed logistic regressions with handling score as a five-modality categorical variable (Online Resource 2).

To investigate the physiological stress response of neonates captured during summer, we fitted a set of linear mixed models explaining variation in cortisol levels as a function of the two-way interaction between log(Ncaptures) and study area, as well as the fixed effects of sex and age (in days), with individual identity as a random factor. Because there is an obvious positive relationship between the age of the neonate and the number of recaptures, we standardized age by taking the residuals of the linear relationship of age with the number of captures experienced by a given individual during summer. Thus, this explanatory variable describes the relative age of the fawn at a given capture event. For each of the three sets of candidate models described above, we compared the most complex models with all simpler nested models using the Akaike’s information criterion corrected for small sample size (AICc) and Akaike weights (*ω*) (Burnham et al. 2011). When several models had a similar level of support (i.e. ΔAICc < 2), we employed the principle of parsimony, retaining the model with the lowest number of parameters (*K*).

Based on the selected linear mixed models, we estimated individual consistency in cortisol level and docility by calculating adjusted repeatability estimates (Nakagawa and Schielzeth 2010). Estimates of adjusted repeatability (*r*) describe the proportion of total variance accounted for by among-individual differences (Bell et al. 2009), while controlling for effects of other covariables (see “Results”). In addition, given the expected relationship between the physiological and behavioral stress responses, we also tested the correlation between individual cortisol level and docility using a Spearman’s rank correlation (*n* = 233 observations).

Finally, given the expected lower stress responsiveness of neonates during the hypo-responsive period, using data from first capture events only, we tested for a difference in cortisol levels between neonates (*n* = 41 individuals) and deer of more than 5-months old (*n* = 50 individuals) using a Student’s *t* test.

All analyses were conducted in R 3.3.2 (R Core Team 2016) using the libraries ‘lme4’ (Bates et al. 2015), ‘MuMIn’ (Bartoń 2016) and ‘ordinal’ (Christensen 2015) to perform and compare models.

## Results

### Stress-induced cortisol response

**Fig. 1:**
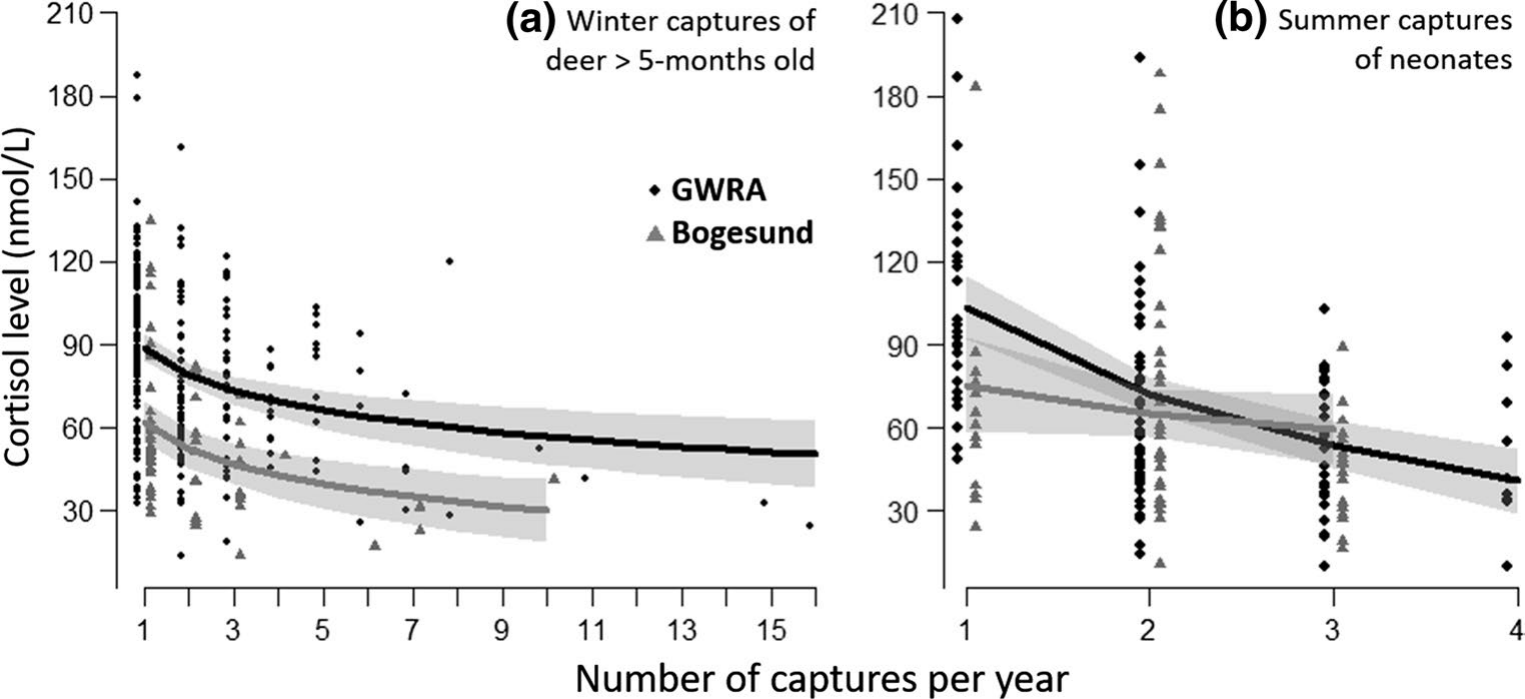
Estimated stress-induced cortisol responses of **a** roe deer of more than 5-months old captured during winter (*n* = 299 observations on 180 individuals) and **b** neonates captured during summer (*n* = 176 observations on 117 individuals), as predicted by the best models, in relation to the number of captures experienced within year and the study area (GWRA vs Bogesund). The grey shadows represent the 95% confidence intervals. Observed values for GWRA (black dots) and Bogesund (grey triangles) were displaced slightly to avoid overlapping

During winter, cortisol levels varied from 14 to 187 nmol/l (mean ± SD 75 ± 30 nmol/l). The selected model indicated that the number of captures within the year and study area were the main additive effects explaining variation in cortisol levels (conditional *R*^2^ = 0.49; see Table 2 and Online Resource 3 for associated *p* values). In particular, we observed a marked habituation in the stress-induced cortisol response which declined as a function of the number of captures within the year, and higher absolute cortisol levels in GWRA compared to Bogesund (predicted estimates varied from 89 to 57 nmol/l and from 62 to 30 nmol/l between the first and the 10th capture at GWRA and Bogesund, respectively; Fig. 1). Although not included in the best model, there was some support for the hypothesis that age explained variation in cortisol levels (ΔAICc = 1.0, AIC weight = 0.10), with old individuals exhibiting a lower cortisol response in comparison with younger individuals (old = 74 ± 5 nmol/l, adult = 85 ± 3 nmol/l, yearling = 86 ± 4 nmol/l and juvenile = 83 ± 3 nmol/l).

**Table 2:** Summary of the candidate linear mixed models for explaining variation in the stress-induced cortisol response and docility level in roe deer of more than 5-months old (winter) and neonates (summer)

Response variable	Models	*K*	AICc	ΔAICc	*ω*
Cortisol response during winter captures	**log(Ncaptures) + Area**	**5**	**2792.6**	**0**	**0.21**
	log(Ncaptures) + Area + Nwinters	7	2794.5	1.9	0.08
	log(Ncaptures) + Area + Age	8	2794.6	2.0	0.08
	log(Ncaptures) + Area + Sex	6	2794.7	2.1	0.07
	log(Ncaptures) × Area	6	2794.7	2.1	0.07
	log(Ncaptures) × Sex + Area	7	2795.0	2.4	0.06
Cortisol response of neonates during summer	**log(Ncaptures)** × **Area + Age**_**S**_	**7**	**1753.1**	**0**	**0.30**
	log(Ncaptures) × Area + Age_S_ + Sex	8	1754.8	1.7	0.13
	log(Ncaptures) × Area + log(Ncaptures) × Age_S_	8	1754.9	1.8	0.12
	log(Ncaptures) + Age_S_	5	1755.7	2.6	0.08
Docility scores during winter captures in GWRA	log(Ncaptures) × Nwinters + log(Ncaptures) × Age + log(Ncaptures) × Sex	16	2417.4	0	0.60
	**log(Ncaptures)** × **Nwinters **+** log(Ncaptures)** × **Age **+** Sex**	**15**	**2418.7**	**1.2**	**0.33**

We tested for the effects of the log-transformed number of captures experienced by a given individual within year (log(Ncaptures)), the number of different winters of capture (Nwinters; for winter captures only), the study area (Area; for cortisol response only), and for sex (Sex) and age (Age, or standardized age Age_S_). *K* is the number of estimated parameters for each model, AICc is the value of the Akaike Information Criterion corrected for small sample size and *ω* is the AICc weight. The retained model is given in bold. Here, we only show models with a ΔAICc < 3 from the best models

During summer, the cortisol level of neonates varied from 10 to 208 nmol/l (mean ± SD 70 ± 39 nmol/l). The selected model included the two-way interaction between the number of captures within the year and study area, as well as standardized age as an additive effect (conditional *R*^2^ = 0.29; Table 2). As above, we found that neonates exhibited higher absolute stress-induced cortisol levels in GWRA, with a marked decrease as a function of the number of captures (from 104 to 54 nmol/l between the first and the third capture), whereas cortisol levels were lower in Bogesund, and, as a consequence, there was almost no variation in relation to the number of captures (from 75 to 60 nmol/l between the first and the third capture). We also observed a decrease in cortisol levels with increasing standardized neonate age (from 109 to 52 nmol/l over the whole range of age; Online Resource 4).

Finally, we found no differences in cortisol levels at first capture between summer neonates and roe deer of more than 5-months old (mean cortisol levels of 93.4 and 86.4 nmol/l, respectively; Student’s *t* test: *t* = 0.89, *p* = 0.38).

### Handling score

Handling scores of roe deer captured during winter varied from 0 to 4 (mean ± SD 1.6 ± 1.0). The selected model included two two-way interactions, between Ncaptures and Nwinters and between Ncaptures and age, as well as the additive effect of sex (conditional *R*^2^ = 0.53; Table 2). The results obtained when the handling score was considered as a five-category factor using ordinal mixed logistic regression were very similar (same model selected, see Online Resource 2 for comparison).

**Fig. 2:**
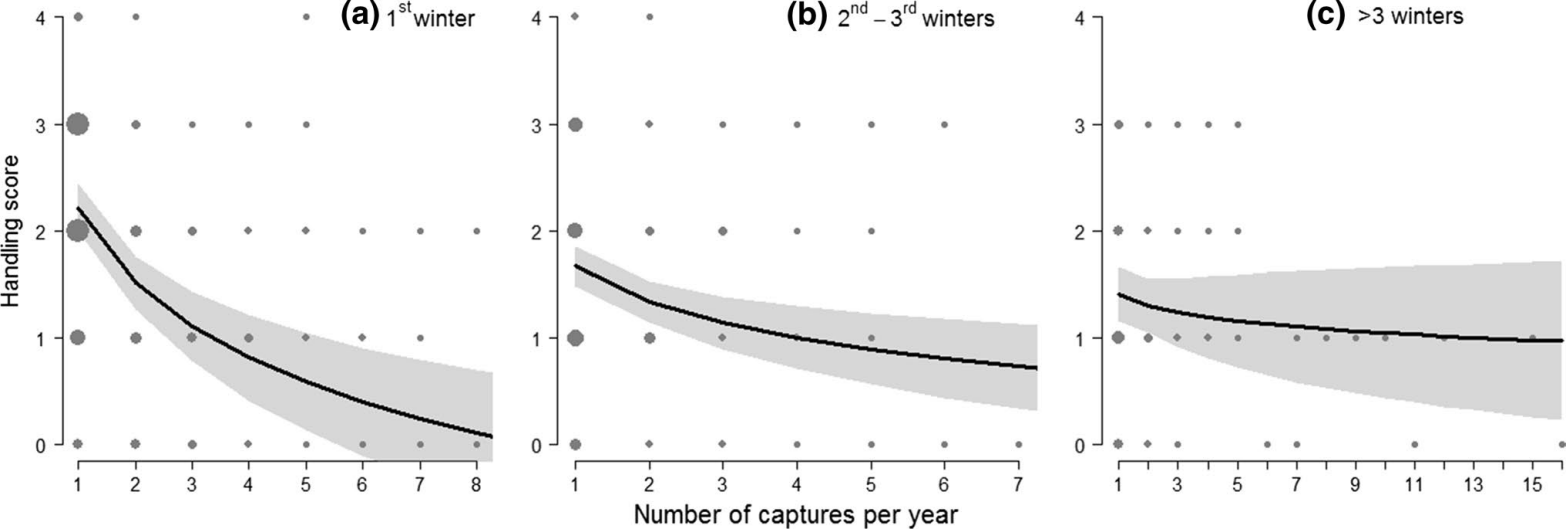
Estimated behavioral response of roe deer of more than 5-months old captured during winter (*n* = 924 observations on 301 individuals), as predicted by the best model describing variation in handling score in relation to the number of captures per year over multiple winters: **a** 1st winter of capture, **b** 2nd and 3rd winters of capture and **c** after more than 3 different winters of capture. The grey shadows represent 95% confidence intervals

As for the cortisol response, there was strong support for habituation, as the handling score markedly decreased with the number of captures within the year, but particularly during the first winter of capture (Fig. 2a). During the following winters, the initial handing score at first capture was lower (Fig. 2b, c) compared to the very first capture. However, the initial behavioral response at first capture in a given winter was always higher than at subsequent captures, suggesting that the level of the stress response recovered partially after several months with no capture stimuli (i.e. 453 days on average). For example, estimated handling score decreased from 2.2 to 0.2 between the first and the 7th capture during the first winter, whereas it declined from 1.4 to 1.1 after the 3rd winter of capture. We also found that old and adult roe deer habituated faster to repeated capture (estimates ranged from 2.3 to 0.5 between the first and 6th capture within a year for both categories) than juveniles and yearlings (from 2.2 to 1.3; Online Resource 5). Finally, females tended to exhibit stronger handling scores than males, indicating that males are slightly more docile (estimated mean ± SD for females: 2.2 ± 0.1 and for males: 2.0 ± 0.1). Although there was some support for an effect of the two-way interaction between sex and log(Ncaptures), the sex difference in slopes for the effect of the number of captures within a winter on handling score was not biologically informative as it was mainly driven by a small difference in score at first capture (i.e. 2.3 ± 0.1 and 1.9 ± 0.1 for females and males, respectively), as previously documented (Debeffe et al. 2015).

### Repeatability and covariation among components of the stress-induced response

While controlling for all covariates featuring in the selected models explaining variation in both cortisol level and handling score, we calculated adjusted repeatability estimates to quantify the residual variance due to among-individual variation. Estimates of adjusted repeatability suggested consistent individual differences in the stress response of roe deer captured during winter, as both cortisol level and handling score were strongly repeatable (*r* = 0.47 ± 0.02 and *r* = 0.35 ± 0.04, respectively). However, in neonates captured during summer, the repeatability estimate for cortisol level was not significantly different from 0 (*r* = 0.04 ± 0.07), suggesting that within-individual variance is more important than the among-individual variance in newborn fawns.

**Fig. 3:**
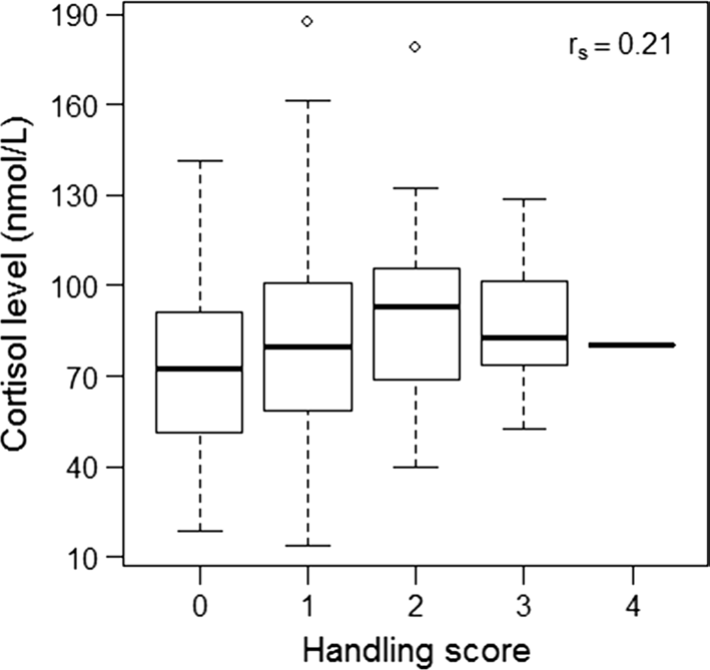
Relationship between cortisol level and handling score in response to stress at capture for individual roe deer of more than 5-months old in GWRA (*n* = 233 observations)

Finally, the cortisol level and handling score were significantly positively correlated across individuals captured during winter at GWRA, although this relationship was not particularly strong (Spearman’s statistic *S* = 1,674,800, correlation estimate *r*_*s*_ = 0.21, *p* value = 0.002). That is, individuals that expressed a higher stress-induced cortisol response also had a higher behavioral score, i.e. were less docile (Fig. 3).

## Discussion

In this study, we investigated the stress-induced physiological and behavioral response of free-ranging roe deer. First, we found that roe deer in the Bogesund population, where human disturbance is frequent, exhibited a lower stress-induced cortisol response than the animals of the GWRA population which are confronted by natural predators, notably lynx and wolf, providing some support for our first hypothesis (**H1**). Contrary to our second hypothesis, we found that neonates exhibited a cortisol response that was as strong as that of adults, suggesting that there is no hypo-responsive period in newborn roe deer (**H2**). To our knowledge, this is the first study describing variation in the stress-induced cortisol response of neonates in a wild mammal. Finally, at the individual level, estimates for repeatability of both cortisol and docility levels suggested that individuals strongly habituated to the stress of handling and differed consistently in how they respond to a stressor (**H3**) such that individuals with a high cortisol response during handling were also less docile, providing further evidence of a stress-management syndrome in roe deer.

1. Between-population differences in the stress response: are natural predators more stressful than humans?

Our results supported our initial expectation, since individuals in GWRA exhibited substantially higher cortisol levels than those in Bogesund (e.g. an absolute difference of 30%, in stress response to the very first capture event; Fig. 1a). However, habituation to humans could also explain this pattern. According to Koolhaas et al. (2011), intense stress occurs in highly uncontrollable, unpredictable and life-threatening situations. Compared to large carnivores, human activities likely have higher temporal predictability, making them easier to foresee and control (for example by avoiding areas used by humans during daytime; e.g. Bonnot et al. 2013). Previous studies on the same populations found that deer in Bogesund were less disturbed by an approaching human than at GWRA (Kjellander and Rooth 1994), suggesting that roe deer are more habituated to human disturbance in Bogesund, whereas they perceive humans as scarier in GWRA.

At GWRA, large carnivores have strongly affected the demography of the roe deer since their return during the 90’s, notably the lynx which is a highly efficient stalk-and-ambush predator (Andrén and Liberg 2015). However, despite the fact that predation is the main mortality cause in this population (Online Resource 1), it seems that lynx have only a limited impact on deer habitat selection (Samelius et al. 2013), suggesting that roe deer may have more difficulty coping with the stress induced by the risk of lynx predation. However, note that, while predation risk has been recognized as a major factor affecting the stress response (e.g. Sheriff et al. 2009), a number of other factors may drive variation in stress levels in wild populations, for example, conspecific density, the level of parasitism and food limitation (Boonstra et al. 1998; Sheriff et al. 2009).

Similarly, we found a marked difference between populations in the stress-induced cortisol response of neonates at the first capture event (absolute cortisol levels were 27%, i.e. 28 nmol/l, higher in GWRA than in Bogesund; Fig. 1b). This higher cortisol response among juvenile and adult roe deer in GWRA could indicate (1) a direct phenotypic adjustment to environmental stressors, or (2) genetic and/or environmental maternal effects. Although, we cannot distinguish between these two (non-exclusive) hypotheses, if maternal effects do occur, they may have profound consequences for offspring phenotype (Kapoor et al. 2006; Love et al. 2013). For example, in free-ranging marmots (*Marmota flaviventris*), Petelle et al. (2017) showed that old mothers with higher cortisol levels produced offspring that were less docile. This type of maternal effect could drive the evolution of persistent differences in the stress response between populations exposed to contrasting stressors (Koolhaas et al. 1999; Bonier et al. 2009; Fischer et al. 2014).

2. Plasticity in the stress response within populations: the role of habituation

We observed marked plasticity in the stress response, as individual roe deer showed strong habituation in both cortisol and behavioral responses to repeated capture (Figs. 1, 2). Overall, short-term habituation resulted in a 53% decrease in stress responses between first and last capture within a given winter. Moreover, we also observed long-term habituation over years, since the behavioral response to capture declined somewhat between successive winters (Fig. 2). By avoiding unnecessary expenditure of time or energy linked to the stress response, habituation to non-threatening stimuli can enhance individual fitness (e.g., Thompson and Henderson 1998; Rodríguez-Prieto et al. 2010). Considering that habituation confers a selective advantage, individuals that are more frequently submitted to mild stressors should habituate faster. A recent study on house sparrows (*Passer domesticus*) showed that urban individuals habituate faster to human disturbance than rural birds, although animals of both populations did not differ in their initial behavioral response (Vincze et al. 2016). Contrary to this study, we did not find strong evidence to suggest that the strength of habituation differed between populations submitted to human disturbance or to predation risk from large carnivores.

However, our study also suggests an effect of age in the strength of habituation, as adults (both prime-age and old individuals) habituated faster than juveniles or yearlings (Online Resource 5). Furthermore, neonates habituated three times faster than older roe deer in GWRA (Fig. 1) and exhibited a capture-induced cortisol response that was similar in magnitude to that of older individuals (Fig. 1). Although this result suggests that there is no hypo-responsive period during early growth in roe deer, an ability to rapidly habituate could protect neonates from the potential long-term deleterious effects of stress (De Kloet et al. 1988; Romero 2004). The much lower rate of habituation for neonates in Bogesund is likely linked to their lower absolute cortisol levels. Although a stress hypo-responsive period has been described in many species (Romero 2004; Lupien et al. 2009), it is as yet unclear whether it occurs widely in natural populations. Variation among-species in the occurrence of a neonatal hypo-responsiveness could be explained by variation in the timing of maturation of the HPA axis (Novais et al. 2017). Indeed, a lack of responsiveness is likely adaptive in precocial species that give birth to relatively immature offspring, in that this protects the developing animal from the harmful side-effects of glucocorticoids (Sapolsky and Meaney 1986). A contrario, this protection is likely unnecessary for altricial species, such as roe deer, that give birth to relatively mature offspring where most brain and neuroendocrine maturation occurs in utero.

3. Among-individual differences within populations: evidence for a stress-management syndrome

Although variation in environmental conditions and habituation seem to strongly drive physiological and behavioral responses, we also observed marked among-individual variations. Indeed, both cortisol and docility levels were strongly repeatable, suggesting that individuals respond consistently to a given stressor. Furthermore, more docile individuals also tended to have a lower cortisol level. These results are coherent with previous studies under both natural and experimental conditions, supporting the existence of a stress-management syndrome in roe deer (Bonnot et al. 2015; Monestier et al. 2016). However, our results contradict the commonly advanced idea that more docile individuals generally exhibit a stronger physiological stress response. Indeed, according to Koolhaas et al. (1999), reactive animals should be more docile, less active and release higher cortisol levels in response to a stressor compared to proactive animals. For example, Martin and Réale (2008) found that cortisol levels in hair samples of free-ranging chipmunks increased with increasing docility. Although coping styles have been widely used to describe among-individual differences in a large range of taxa (Koolhaas et al. 1999), the relationships among behavioral and physiological stress responses are not well understood. Koolhaas et al. (2007) suggested that a two-tier model, combining a coping style axis indexing the quality of the behavioral response (e.g., aggressiveness, activity) with an emotionality axis indexing the magnitude of the stress response (e.g. cortisol level) might provide a better description of how individuals cope with stress. According to this model, we speculatively suggest that roe deer with a low cortisol level and a low behavioral response to stress are “docile”, individuals with a high cortisol level and high behavioral response are “panicky”, whereas individuals with a high cortisol level, but a low behavioral response could be categorized as “shy” (Fig. 3).

## Conclusion

Both stress-induced and baseline cortisol levels are generally considered to be contingent on long-term hypertrophy of the adrenal gland, resulting in stronger stress responsiveness (Clinchy et al. 2004; Sheriff et al. 2010). In this study, we estimated the stress-induced cortisol level in response to handling, but not the baseline cortisol level. The marked difference (approximately 30%) observed in the stress-induced cortisol response between the two roe deer populations may have profound consequences in terms of individual fitness. Indeed, prolonged exposure to stressors (such as cues of predator presence), can have long-term impacts on reproductive success and survival in free-ranging animals (Preisser et al. 2005; Zanette et al. 2011; Clinchy et al. 2013).

Both our estimates of repeatability and an observed among-population difference in naïve neonates suggest that genetic and/or environmental maternal effects sustain individual variation in stress response. Because variation in both physiological and behavioral responses may be involved in generating and maintaining consistent personality differences, future studies should investigate whether among-individual differences in the stress response translate into variation in individual fitness or life-history strategy (Smith and Blumstein 2008; Bonier et al. 2009; Réale et al. 2010). In roe deer, the position along the shy-bold gradient influences the way an individual exploits habitats of contrasting risk (Bonnot et al. 2015), with consequences for annual reproductive success (Monestier et al. 2015). Although we might expect that relationships between individual variation in stress responses and fitness are widespread, more studies are needed to better understand the consequences of stress in wild populations (Clinchy et al. 2004, 2013; Reeder and Kramer 2005; Bonier et al. 2009), particularly in the context of the recovery of large natural predators across Europe (Chapron et al. 2014).

## Electronic supplementary material

Below is the link to the electronic supplementary material.

Supplementary material 1 (PDF 166 kb)
