# Supplementary material for: Who’s afraid of the big bad wolf? Variation in the stress response among personalities and populations in a large wild herbivore
Source: Oecologia. 2018 May 26;188(1):85–95. doi: 10.1007/s00442-018-4174-7 (PMC6096777; doi:10.1007/s00442-018-4174-7)

ONLINE RESOURCES

Who's afraid of the big bad wolf? Variation in the stress response among personalities and populations in a large wild herbivore

Nadège C. Bonnot[1*], Ulrika A. Bergvall[1,2], Anders Jarnemo[3] & Petter Kjellander[1]

*Corresponding author: nadege.bonnot@slu.se; +46702467321

Online Resource 1:

Table summarizing the proportion of mortality events for the main causes of mortality, namely Hunting, Natural causes (i.e. diseases or starvation), Road traffic accident, Predation by lynx or wolf, Predation by fox (only for neonates) and Other/Unknown causes (other causes like "gored" for adults or "mowing" for neonates, but mainly unknown causes of death). Between 2010 and 2014, 131 and 57 mortality events were recorded in GWRA and Bogesund, respectively. For comparison, note that 190 and 210 adult roe deer were captured respectively during winter within this period. Between 2013 and 2016, 101 and 117 neonates were captured and monitored throughout the summer, and 46 and 60 mortality events were recorded in GWRA and Bogesund, respectively. Although more information is available in the most recent years, we decided to not include this in this table because the monitoring effort was unbalanced between the two populations, potentially leading to biased estimates of the risk of death among the different mortality causes.

**Mortality causes**	**Adult mortality**		**Neonate mortality**	
	GWRA (N=131)	Bogesund (N=57)	GWRA (N=41)	Bogesund (N=60)
Hunting	10%	47%	-	-
Natural	14%	26%	12%	5%
Road traffic accident	5%	7%	5%	-
Predation (lynx, wolf)	49%	2%	22%	-
Predation (fox)	-	-	29%	23%
Predation (undetermined or other predators)	10%	2%	15%	42%
Other/Unknown	12%	16%	17%	30%

Online Resource 2: *Summary and results of the ordinal mixed logistic regressions on the 5-modality factor for handling score.*

Although we used docility score as a continuous variable representing a gradient in the behavioural response of roe deer to capture stress in the main manuscript, the handling score could also be viewed as a categorical variable with five ordered modalities ($0 < 1 < 2 < 3 < 4$). Therefore, for comparison, we also performed ordinal mixed logistic regression models on the 5-modality handling score.

As described in the main text, we controlled for the effects of sex and age (4 classes: juvenile of 5-10 months old, yearling of 1.5 years old, adult of 2.5-6.5 years old and old individuals of 7.5-12.5 years old) in the analyses, because intrinsic factors may modulate the response of an individual to a given stressor. To investigate habituation to repeated capture events over both the short and long-term, we determined the number of captures experienced by each individual within a year (Ncaptures, short-term habituation) and the number of different winters during which each individual was captured (Nwinters, long-term habituation). Because we did not expect long-term habituation to be governed by a linear effect, we included Nwinters as a categorical variable with three modalities: first winter of capture (1st winter), second and third winters of capture (2nd-3rd winters) and more than three winters of capture (> 3 winters). Because animals can habituate to repeated stressors at both the short- and long-term, we included the two-way interaction between the log-transformed number of captures experienced by an individual within a year (log(Ncaptures)) and the number of different winters of capture (Nwinters). To account for intrinsic characteristics of individuals, we also included sex and age, as well as their two-way interactions with log(Ncaptures). Therefore, the most complex model explaining variation in handling score included the four two-way interactions between log(Ncaptures) and, respectively, Nwinters, age and sex. Finally, we also included individual identity as a random factor on the intercept in all models to control for repeated observations of individuals.

We compared this most complex model with all simpler nested models using the Akaike's information criterion corrected for small sample size (AICc) and Aikaike weights (ω) (Burnham et al. 2011). All analyses were conducted in R 3.3.2 (R Core Team 2016) using the libraries 'ordinal' (Christensen 2015) and 'MuMIn' (Bartoń 2016) to perform and compare ordinal mixed logistic regression models.

The results obtained with ordinal mixed logistic regression models were very similar to those presented in the main manuscript using linear mixed models on the continuous docility score. Notably, the models selected by AICc were similar for both analyses (see Tables below and Table 2 in the main manuscript). That is, the selected model included three two-way interactions between the number of captures within the year with the number of winters of capture, age, and sex.

Table of the candidate ordinal mixed logistic regressions for explaining variation in docility level in roe deer of more than 5-months old captured during winter. We tested for the effects of the log-transformed number of captures experienced by a given individual within winter (log(Ncaptures)), the number of different winters of capture (Nwinters), sex and age. K is the number of estimated parameters for each model, AICc is the value of the Akaike Information Criterion corrected for small sample size and ω is the AICc weight. The retained model is given in bold. Here, we only show models with a ΔAICc < 3 from the best models.

**Response variable**	**Models**	**K**	**AICc**	**ΔAICc**	**ω**
Docility score as a *5-modality ordered* factor	**log(Ncaptures) x Nwinters + log(Ncaptures) x Age + log(Ncaptures) x Sex**	**18**	**2279.5**	**0.0**	**0.47**
	log(Ncaptures) x Nwinters + log(Ncaptures) x Age + Sex	17	2279.7	0.3	0.41
	log(Ncaptures) x Nwinters + log(Ncaptures) x Age	16	2141.6	2.9	0.37

Table of the parameter estimates with standard errors, z-values and p-values for variables included in the retained model.

Parameters	Estimate ± s.e.	z-value	p-value
log(Ncaptures)	-1.5 ± 0.3	-5.8	<0.001***
Nwinters : 2nd-3rd winters	-1.5 ± 0.3	-4.7	<0.001***
Nwinters : >3 winters	-2.1 ± 0.4	-5.2	<0.001***
Sex: Male	-0.8 ± 0.3	-3.0	0.003**
Age: yearling	0.2 ± 0.4	0.4	0.67
Age: adult	0.1 ± 0.3	0.2	0.84
Age: old	0.6 ± 0.5	1.2	0.24
log(Ncaptures) x Nwinters: 2nd-3rd winters	1.6 ± 0.5	3.2	0.002**
log(Ncaptures) x Nwinters: >3 winters	2.5 ± 0.6	4.2	<0.001***
log(Ncaptures) x Sex: M	0.5 ± 0.3	1.7	0.09
log(Ncaptures) x Age: yearling	-0.3 ± 0.6	-0.6	0.55
log(Ncaptures) x Age: adult	-1.6 ± 0.5	-3.3	0.001**
log(Ncaptures) x Age: old	-1.4 ± 0.7	-2.1	0.03*

Online Resource 3:

Table of the degrees of freedom (df), F-statistics (F) and p-values (p) for each term included in the retained model for each response variable.

**Response variable**	**Model terms**	**df**	**F**	**p**
Cortisol response during winter captures	log(Ncaptures)	1	31.6	<0.0001***
	Area	1	42.8	<0.0001***
Cortisol response of neonates during summer	log(Ncaptures)	1	36.2	<0.0001***
	Area	1	1.0	0.33
	Age_S	1	17.4	<0.001***
	log(Ncaptures) x Area	1	5.1	0.03*
Docility scores during winter captures in GWRA	log(Ncaptures)	1	59.9	<0.0001***
	Nwinters	2	22.3	<0.0001***
	Age	3	2.7	0.046*
	Sex	1	6.7	0.010*
	log(Ncaptures) x Nwinters	2	6.8	0.001**
	log(Ncaptures) x Age	3	4.8	0.003**
	log(Ncaptures) x Sex	1	3.3	0.072·

Levels of significance: *** <0.001, ** <0.01, * <0.05, · <0.1

Online Resource 4: *Estimated cortisol response of roe deer neonates, as predicted by the best model, in relation to standardized age (i.e. age in days, corrected for the number of captures experienced during summer), with 95% confidence intervals (grey shadow). The observed values for GWRA (black dots) and Bogesund (grey triangles) are depicted.*

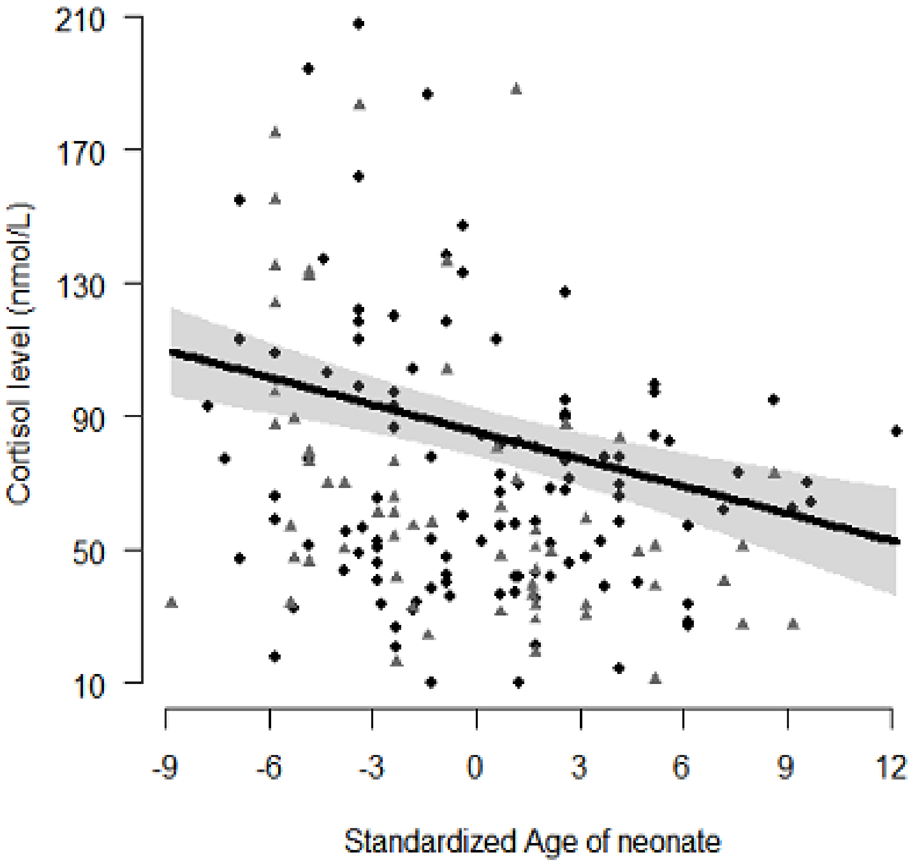

Online Resource 5: *Estimated handling scores of roe deer of more than 5-months old captured during winter, as predicted by the retained model, in relation to the number of captures per year and age categories: juvenile, yearling, adult and old animals. The shadows represent 95% confidence intervals.*

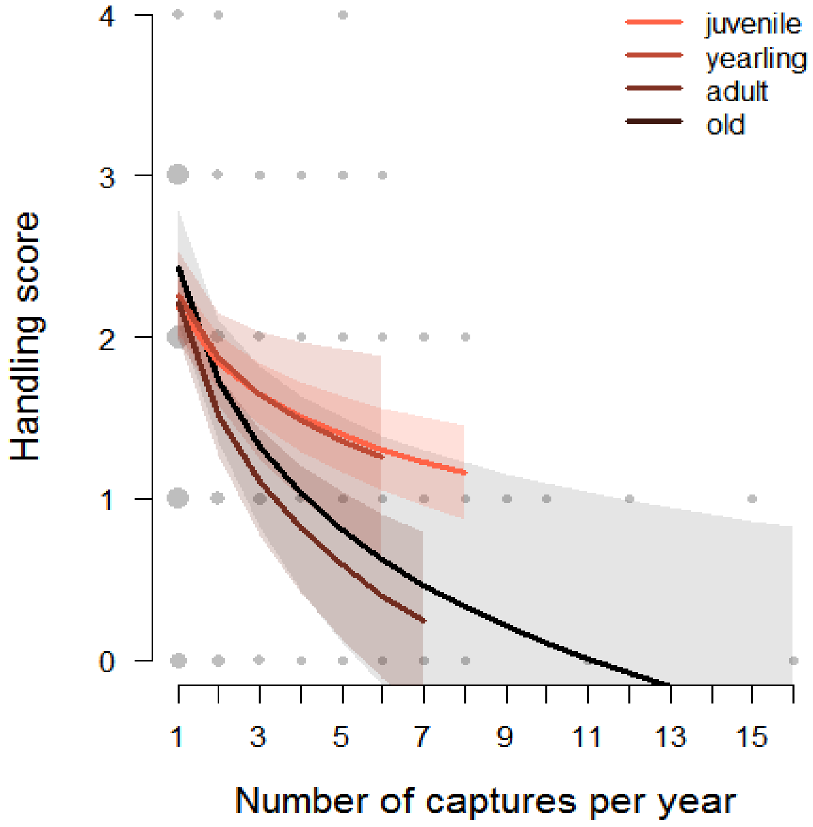

Supplement: Supplementary file 1 — Supplementary material 1 (PDF 166 kb) [file 442_2018_4174_MOESM1_ESM.pdf]
